# The Role of the Assembly Force in the Tribocorrosion Behaviour of Hip Implant Head-Neck Junctions: An Adaptive Finite Element Approach

**DOI:** 10.3390/bioengineering9110629

**Published:** 2022-11-01

**Authors:** Khosro Fallahnezhad, Mohsen Feyzi, Reza Hashemi, Mark Taylor

**Affiliations:** Medical Device Research Institute, College of Science and Engineering, Flinders University, 1284 South Road, Clovelly Park, SA 5042, Australia

**Keywords:** hip implants, assembly force, tribocorrosion, fretting corrosion, finite element, material loss, metallic interface

## Abstract

The cyclic loading, in the corrosive medium of the human body, results in tribocorrosion at the interface of the head-neck taper junction of hip implants. The resulting metal ions and wear debris adversely affect the local tissues. The force applied by surgeons to assemble the junction has proven to play a major role in the mechanics of the taper junction which, in turn, can influence the tribocorrosion damage. Recently, finite element method has been used to predict the material loss at the head-neck interface. However, in most finite element studies, the contribution of electrochemical corrosion has been ignored. Therefore, a detailed study to investigate the influence of the assembly force on the tribocorrosive behaviour of the head-neck junction, which considers both the mechanical and chemical material removal, is of paramount interest. In this study, a finite-element-based algorithm was used to investigate the effect of assembly force on the tribocorrosion damage at the junction interface, for over four million cycles of simulated level gait. The patterns of the material removal in the modelling results were compared with the damage patterns observed in a group of retrieved modular hip implants. The results of this study showed that for different cases, chemical wear was in the range of 25–50% of the total material loss, after four million cycles. A minimum assembly force (4 kN for the studied cases) was needed to maintain the interlock in the junction. The computational model was able to predict the damage pattern at the retrieved head-neck interface.

## 1. Introduction

Modular junctions are widely used in orthopaedic devices, to provide flexibility in restoring joint alignment during surgery. The head-neck taper junction of total hip replacement is a typical example. Early monoblock hip implants were designed for total hip replacement (THR) in the 1960s. Although they have shown good long-term survivorship and clinical outcomes, significant limitations remained in fine-tuning leg length and offset in THR. Hence, since the 1980s, hip joint implants with a modular design of the femoral component have become popular. However, the cyclic loading inside the corrosive medium of the human body results in tribocorrosion (fretting corrosion) at the interface of the head-neck junction, so-called taperosis [1,2,3,4]. The resulting debris and released metal ions have been associated with lytic lesions and pseudotumours, and are known to damage both the cell’s body and the production of free radicals that are associated with various pathologies [5]. Hence, several studies have investigated the mechanism of the material removal at the head-neck junction of hip implants, and the parameters that may influence it [6,7,8]. Due to the complex geometry and multivariable nature of this process, finite element analysis is an effective method for such an investigation. Recently, FE method has been used to predict the material loss at the head-neck interface [9,10,11].

The force applied by surgeons to assemble the head-neck junction is reported to range from 3000 to 7000 N [12]. This force has proven to play a major role in the mechanics of the taper junction which, in turn, can influence the tribocorrosion damage [10]. To date, studies have investigated the influence of the assembly force on the initial mechanical behaviour of the taper junction [13,14,15]. These studies generally suggest that high assembly forces enhance the initial stability and fixation in the head-neck junction, and are therefore able to better withstand mechanical loads of daily activities without disconnection.

In comparison, only a few researchers have taken a step forward and investigated the influence of the assembly force on both the fretting wear that occurs at the taper interface and its associated material removal [9,10,13,16]. English et al. [16] used an adaptive numerical approach to investigate the influence of the assembly force on fretting wear in the head-neck junction. They used an energy-based wear law within a FE framework to simulate fretting wear in a CoCr/Ti-6Al-4V junction, which was subjected to several million cycles of gait loading. Their results showed that the use of higher assembly forces reduces the fretting wear material loss in the head-neck junction.

To further improve the estimation of the material loss during the operation of the hip implant, the tribocorrosion (fretting corrosion) phenomenon at the head-neck junction requires investigation. To achieve this, both the mechanical wear and electrochemical corrosion need to be modelled simultaneously [17]. Modelling tribocorrosion has been a major challenge for researchers, due to its complexity [18]. Several analytical expressions have been proposed by researchers to predict tribocorrosion for simple ball-on-disk configurations [19]. Mischler and Landolt [20,21] proposed a mechanistic approach to estimate the chemical wear, by describing the anodic current as a function of the passivation charge. This model, in parallel with a simplified Archard wear model, was successfully used by Maldonado et al. [22] to quantify mechanical and chemical volume losses for a CoCrMo alloy in a ball-on-disk tribocorrosion test.

To predict the tribocorrosion process for complex geometries, and the loading configurations of real engineering applications, several researchers have tried to use the analytical tribocorrosion models within numerical frameworks [19].

Recently, the authors have developed a finite element model to simulate the tribocorrosion process to predict material loss due to both the mechanical fretting wear and the passivation [23,24]. The model used a combination of Landolt’s passive film equation [20] together with Archard’s wear law and was successfully validated by existing experimental tests [24]. This provided a foundation for further analysis on the influential parameters in hip replacements surgeries.

To date, no predictive study, which considers both the mechanical (substrate removal) and chemical (oxide removal) material losses, has been carried out to investigate the influence of the assembly force on the tribocorrosive behaviour of the head-neck junction of the hip implant. In this work, the developed tribocorrosion algorithm [24] is employed and used to investigate the influence of the assembly force on the tribocorrosive behaviour of a CoCr/CoCr head-neck junction.

## 2. Methodology

### 2.1. FE Tribocorrosion Model

The details of the tribocorrosion model and the steps in the prediction of the volume loss are given in the authors’ previous work [24]. Briefly, a combination of Landolt’s passive film equation [20] and Faraday’s law [19] (Equation (1)), in parallel with Archard’s wear model [25] (Equation (2)), was used within a FE model to simulate the fretting corrosion phenomenon in this study. The chemical corrosion material loss (*V_chem_*) is described by Equation (1).

(1) $$V_{chem}=N\cdot \frac{K_{a}\cdot v_{s}\cdot Q_{p}\cdot {\left(\frac{F_{N}}{H}\right)}^{0.5}\cdot t_{rub}\cdot M}{n\cdot F\cdot \rho }=N\cdot K_{a2}\cdot Q_{p}\cdot d\cdot \sqrt{\frac{F_{N}}{H}}\;;\;K_{a2}=\frac{K_{a}\cdot M}{n\cdot F\cdot \rho }$$

where *K_a_* is a proportionality constant that depends on the number of acting asperity contacts and their probability to depassivate the metal, vs. is the sliding velocity, *Q_p_* is repassivation charge density, *F_N_* is the normal force, *H* is the indentation hardness, *t_rub_* is rubbing time, *M* is molecular mass, *n* is charge number, *F* is Faraday’s constant and *ρ* is the mass density, *K_a2_* is the chemical wear coefficient, *d* is the rubbing distance and *N* is the update interval that will be explained later.

Equation (2) represents the Archard law, which is used in this study to model the mechanical wear mechanism.

(2) $$\frac{V_{mech}}{d}=N\cdot k\frac{F_{N}}{H}$$

where *V_mech_* is the mechanical lost volume, *d* is the sliding distance, *k* is the wear coefficient, *F_N_* is the normal load and *H* is the material hardness. This equation has been widely used by researchers to predict the material removal in both wear and fretting wear mechanisms [26,27,28].

*K_a2_* and *k*, for the CoCrMo alloy were determined to be 1.89 × 10^−12^ C/cm^2^ and 2.79 × 10^−12^, respectively, based on experimental data [22].

**Figure 1:**
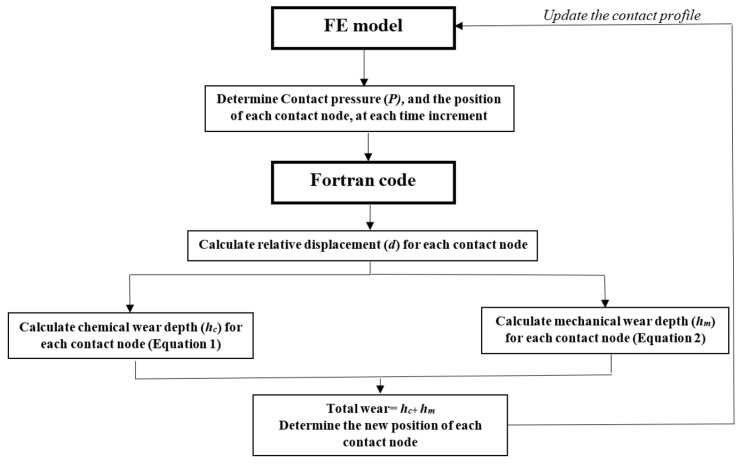
The algorithm of the FE model and FORTRAN code.

To model fretting corrosion, a FORTRAN code was developed to control the position of the contact nodes. The algorithm of the FORTRAN code and FE model, which is applied to the surface nodes at each time increment, is given in Figure 1. Changing the position of the contact nodes changes the contact profile; accordingly, this changes the magnitude of the contact pressure (*P*) and relative displacement for each node. The new values of chemical and mechanical material loss are calculated by the FORTRAN code and are then applied to the contact elements of finite element model, at each time increment. Such an adaptive process helps to simulate tribocorrosion more accurately, considering the influence of “contact profile changing” on the tribocorrosion simulation process. This algorithm calculates the mechanical and chemical wear components for each contact node and updates the position of the nodes, considering the synergetic effect between wear and corrosion. The simulation of every load cycle is computationally expensive. In order to reduce the computational cost, an update interval (*N*) can be used which assumes that the wear rate remains constant during a pre-determined number of cycles. In previous studies [11,16,29], this factor was selected as 10^5^ cycles. According to a convergency study in the authors’ previous work, 200,000 was selected as the most optimized value for N.

In the authors’ previous work [24], the developed tribocorrosion FE algorithm was successfully verified by replicating a set of experimental, reciprocating ball-on-disk tribocorrosion tests.

### 2.2. Head-Neck Finite Element Model

**Figure 2:**
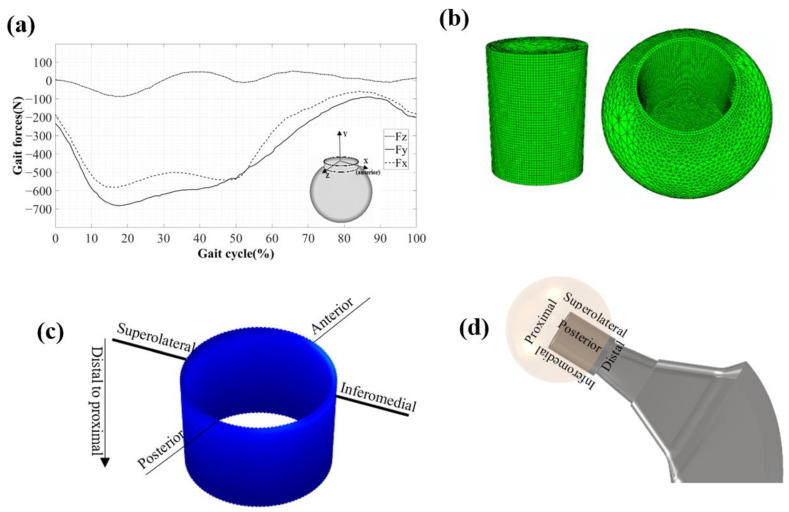
(**a**) Gait force profiles; (**b**) Meshing structure of the head-neck FE model; (**c**) Different regions in the head and neck surfaces; (**d**) Schematic view of the head-neck junction of a hip implant.

A 3D model of a 32-mm diameter CoCr head (with a conical angle 2.83°) and a 12/14 CoCr neck (with a height of 17.7) [30] was modelled under normal walking load profiles [31]. The head and the neck have a distal angular mismatch of 0.01°. An elastic-linear plastic material model was used for CoCr (ISO 5832-12) with: a Young’s modulus of 210 GPa; Poisson’s ratio of 0.30; yield stress of 910 Gpa; ultimate tensile strength of 1350 Gpa; and tensile elongation of 15%. In this model, the outer surface of the head was fixed to have no motion. To appropriately simulate both the contact pressure and the relative displacement, brick elements with an edge size of 0.1 mm were used at the head-neck interface (refined several times to achieve a converged solution) (Figure 2b). The rest of the model was meshed using tetrahedral elements with an increased edge size achieved by distancing from the interface [24]. The FE model of junction consisted of 505,548 elements. The simulation was performed in two stages. In the first stage, the junction was assembled using the assembly force applied to the bottom face of neck (this was a quasi-static force as is detailed in [30]). In the second stage, the developed adaptive FE algorithm was used to simulate fretting corrosion phenomenon in the interface of the head-neck junction, which was subjected to gait loading for four million cycles. The joint reaction components for level gait were derived from [32] (Figure 2a,b) and again applied to the distal surface of the neck. The equation proposed by Bao et al. [33] was used to determine the passivation charge of the head-neck junction. This equation defines the passivation charge for a CoCr-CoCr combination as a function of the working potential independent of the solution type. The working potential of the CoCr head-CoCr neck junction subjected to gait loading has been reported by Farhoudi [31]. Based on his experimental results (using a custom head-neck junction simulator), while the junction was subjected to a gait loading profile with a maximum peak of 900 N, the working potential was −0.25 V for an Ag/AgCl reference electrode. This was converted to the equivalent potential for the Standard Hydrogen reference electrode (−0.045 V), for which Bao’s equation was developed. This potential was then used in Bao’s equation to determine the passivation charge density in the head-neck junction (0.71 mc/cm^2^). Such an approach was used and verified by Cao et al. [34] to determine the passivation charge density for a head-cup interface in a hip-joint testing simulator.

This FE approach was used to predict the fretting corrosion damage at the head-neck junction for assembly loads of 2, 4, 6 and 8 kN. The mechanical and chemical volume losses for each case were determined, at different stages of loading cycles, and up to four million cycles. The contact pressure and the micromotion of the contact nodes were also determined for each case as a function of the number of loading cycles, to better show the influence of assembly force on the mechanical behaviour of the junction. Figure 2c,d show the regions in the head-neck surfaces and schematic view of the head-neck junction of the hip implant, respectively.

### 2.3. The Method of Comparing FE Predicted Tribocorrosive Wear Profiles with Damage Patterns in Retrieved Tapers

A group of modular hip implants (30 samples) were accessed from the orthopaedic implant retrieval library at the Royal Adelaide Hospital (Adelaide, Australia). The head-neck junctions of these implants appeared to be distally contacted and they had similar damage patterns at their interface. The junctions were composed of CoCr head and a 12/14 CoCr neck taper. Other information such as the mismatch angle of the junction, the original assembly condition and the loading history were not available; however, the implants could be generally classified into two distinguishable categories based on their damage patterns/degrees. The damage patterns observed in these categories were similar to the FE-predicted patterns that occurred under 2 kN (Category 1) and above 2 kN (4/6/8 kN) (Category 2) of assembly forces. Therefore, it was assumed they might have been originally assembled by these force levels. Their interface was scrutinised and photographed for a general comparison with the FE results.

## 3. Results

### 3.1. Finite Element

**Figure 3:**
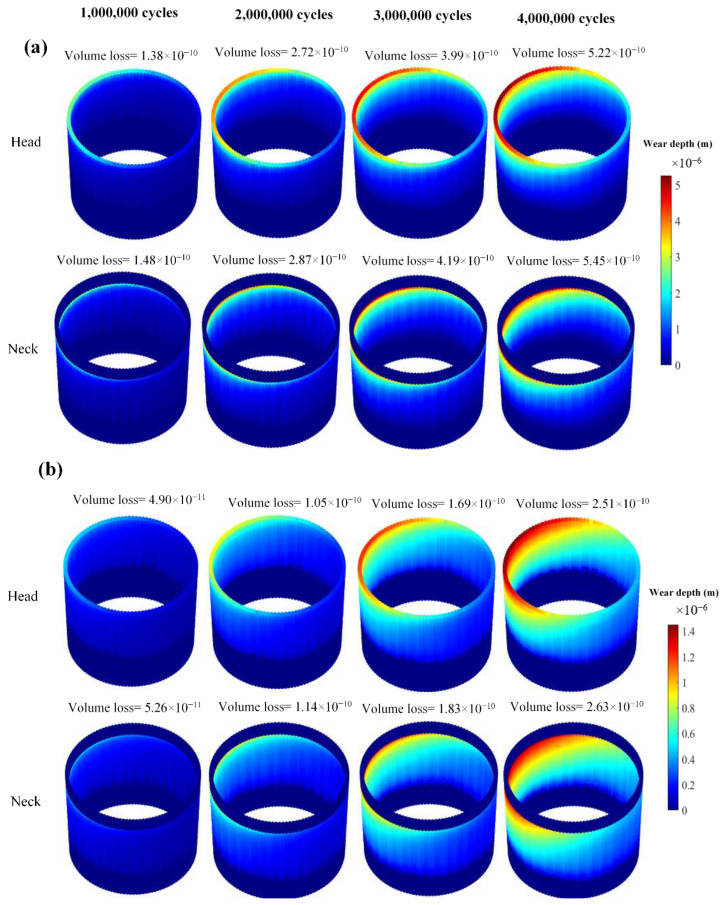
Wear profiles of the head and neck contact surfaces: (**a**) Mechanical; (**b**) Chemical (the wear results are presented in meter).

Figure 3 compares the difference between mechanical/chemical wear in the head and neck for the case with an assembly force of 4 kN. It is obvious from this figure that the head and neck have the same amount of material loss with a negligible difference. The same trend has been seen for the other assembly forces. Hence, to avoid redundancy, the remainder of the paper is only focused on the results of the neck.

**Figure 4:**
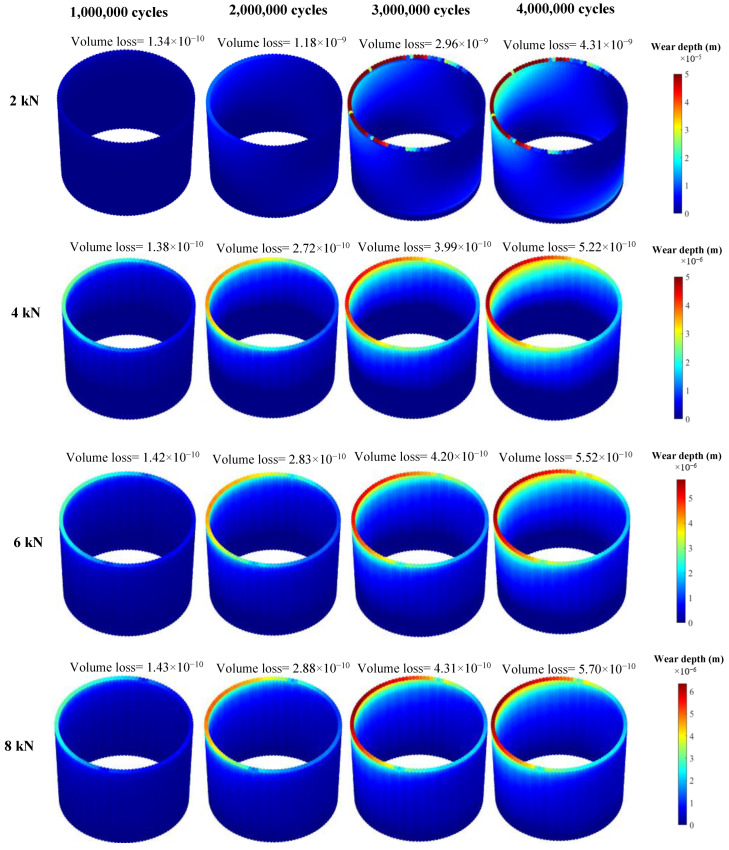
Mechanical wear profiles of the head contact surface (the wear results are presented in meters and the volume losses are in m^3^).

**Figure 5:**
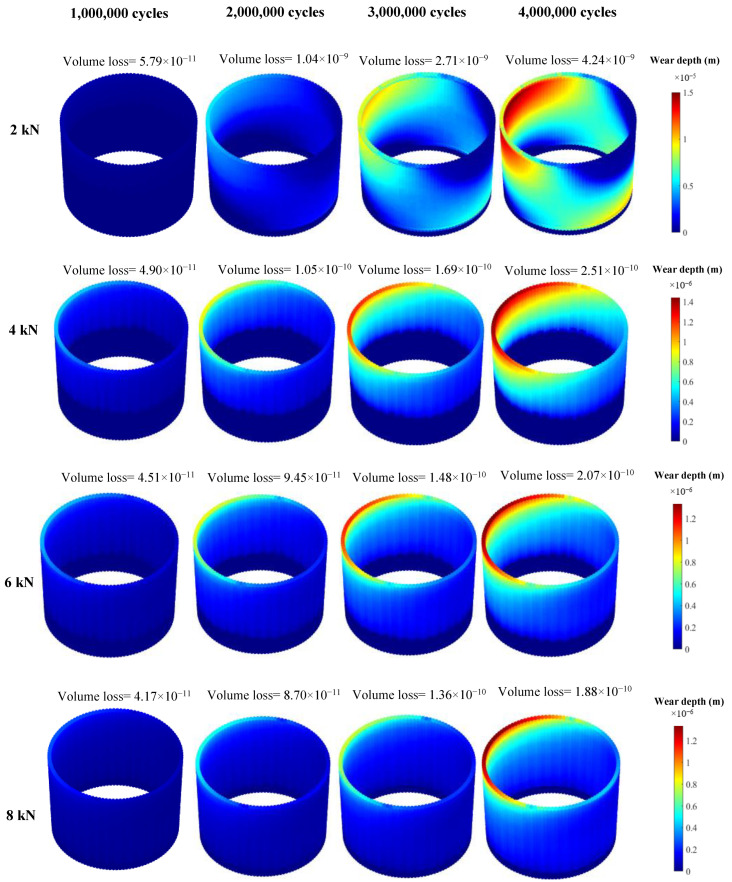
Chemical wear profiles of the head contact surface (the wear results are presented in meter and the volume losses are in m^3^).

Figure 4 and Figure 5 show the mechanical and chemical wear profiles for all four assembly forces. It should be noted that the range of the colour bars are different for different cases to better show the distribution of the variables on the neck surface. Figure 4 clearly shows that the case assembled by 2 kN has the highest mechanical wear at the end of the process, with a volume loss of 4.31 × 10^−9^ m^3^. The 4, 6 and 8 kN cases have the same volume of material loss of 5.22 × 10^−10^, 5.52 × 10^−10^ and 5.70 × 10^−10^ m^3^, after four million cycles. This indicates that the increase in assembly force, from 4 to 8 kN, has a negligible influence on the amount of the material loss caused by mechanical wear. More interestingly, all the cases have almost the same volume loss until one million loading cycles. Halfway through the process, the 2 kN case shows significantly more mechanical wear damage, compared to the other cases. The trend of the chemical wear variation is similar to that of mechanical wear (Figure 5). However, the difference between 2 kN case and other cases is more significant in chemical wear. The 2 kN case has the chemical wear of 4.2 × 10^−9^ m^3^ after four million loading cycles, while 4, 6 and 8 kN cases have volume losses of 2.51 × 10^−10^, 2.07 × 10^−10^ and 1.88 × 10^−10^ m^3^, respectively. Moreover, unlike mechanical wear, the difference between the chemical wear of the 4 kN case and the 6 and 8 kN cases is not insignificant.

**Figure 6:**
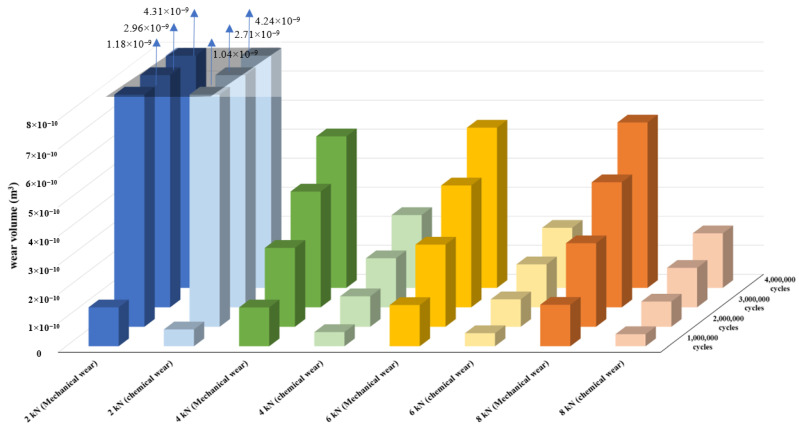
Variation of the mechanical and chemical wear components.

When examining the variation of the mechanical and chemical wear components during the four million cycles of gate loading (Figure 6), it is clear that the 2 kN case has significantly higher wear volumes compared to the other cases. However, this difference is not considerable until the end of the one million loading cycles for both mechanical and chemical wear components. For the 4, 6 and 8 kN cases, the mechanical damage is significantly (more than 2-fold) larger than the chemical wear. Interestingly however, in the 2 kN case, the chemical and mechanical damages have close values of material losses, after two million cycles.

**Figure 7:**
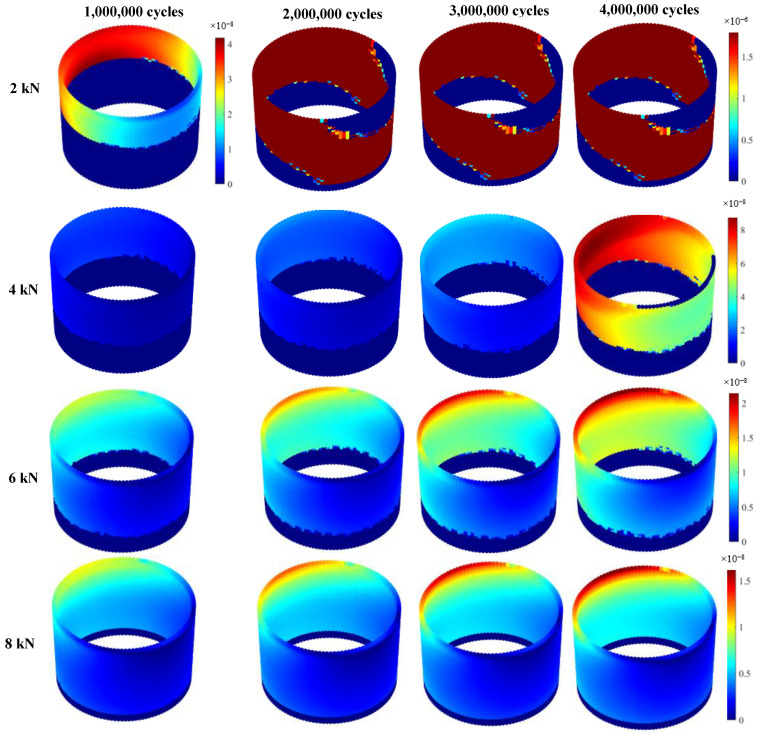
Micromotion profiles of the head-neck interface (Micromotions are presented in meter).

**Figure 8:**
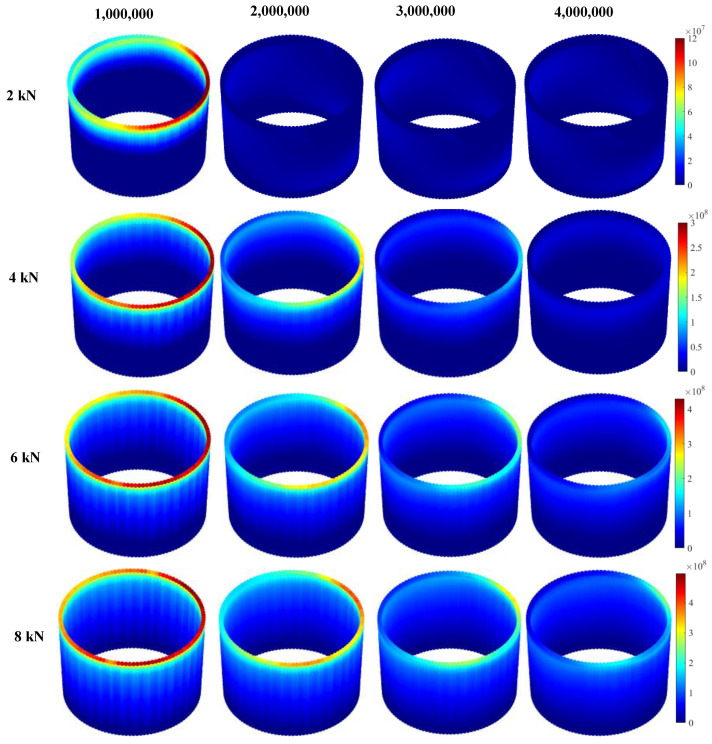
Pressure profiles of the head-neck interface (contact pressures are presented in Pascal).

Contact pressure and relative micromotion at the interface of the head-neck junction play the most important roles in tribocorrosive damage of metallic interfaces. The micromotion profiles show interesting trends for all cases, during the process of simulation (Figure 7). As was expected, the 2 kN case has the largest micro-motion. For this case, the largest micromotion has occurred within the superolateral region, after one million loading cycles, where the maximum micromotion increases to 4.2 × 10^−2^ µm. Following two million cycles of loading, the micromotion for this case results in an extreme increase (up to 640-fold). Such a significant jump is likely, due to the loss of the taper interlock. For the other cases, the maximum micromotion occurs in the superolateral region, and shows an increasing trend. For the 2 kN case, the contact pressure becomes very insignificant (8 MPa) after one million cycles (Figure 8). This confirms the loss of the taper interlock at this stage. For the other cases, the maximum contact pressure exists at the distal end of the junction (all around the distal area). During the simulation process, the contact pressure reduces, particularly within the superolateral region, where the maximum wear occurs.

**Figure 9:**
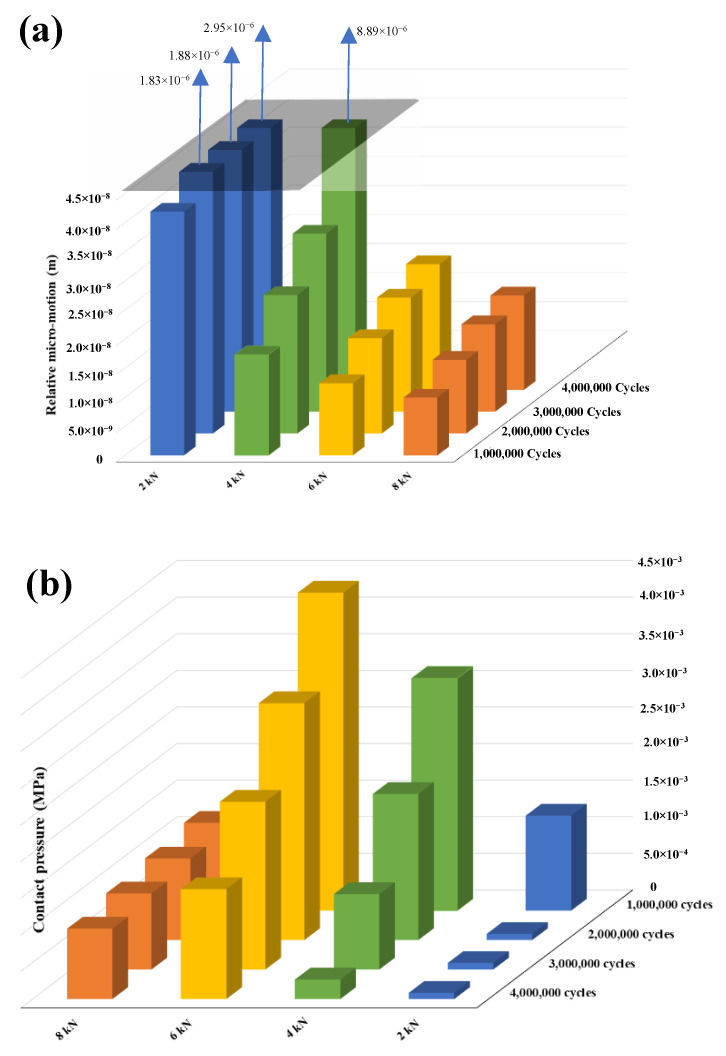
Variation of the: (**a**) Micromotion; (**b**) Pressure, at the neck contact surface.

The maximum micromotion of the 4, 6 and 8 kN cases gradually increases during the four million loading cycles (Figure 9). Such gradual behaviour can also be seen in the contact pressure of these cases. The contact pressure for these three cases monotonically decreases over the simulation process. For the 2 kN case, however, the loss of the interlock results in an extreme reduction of the contact pressure and a remarkable increase in the micromotion, after one million cycles.

### 3.2. Comparing FE Predicted Tribocorrosive Wear Profiles with Damage Patterns in Retrieved Tapers

**Figure 10:**
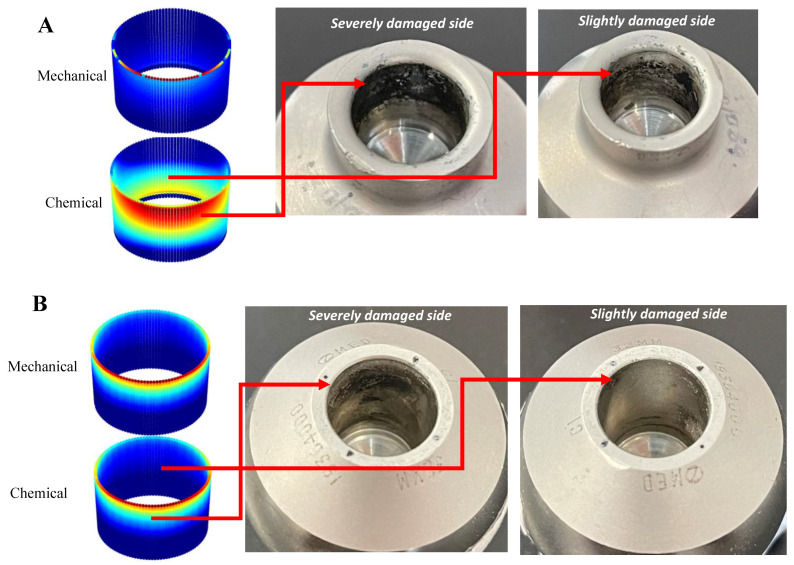
The figure shows comparison between the tribocorrosive wear profiles on the head tapers between FE model and retrieved samples with different levels of assembly force: (**A**) less than/equal to 2 kN, (**B**) more than/equal to 4 kN.

The computational model is able to predict the damage pattern at the head-neck interface. For the first category (assumed to be assembled by less than/equal to 2 kN of force) (Figure 10A), severe damage is observed at the interface. On one face of the interface, this severe damage starts from the taper’s distal end and extends towards its proximal end; however, the damage trace finishes before reaching the proximal end. On the opposite face, less severe damage develops from a location with an initial distance from the distal end and covers a significant proportion of the face. This pattern is similar to that predicted by the FE model (specifically the chemical wear pattern) in which the interlock was lost. Less damage is observed at the interface for the second category (assumed to be assembled by more than/equal to 4 kN of force) (Figure 10B). This level of force seems to restrict the damage severity to one face of the taper; compared to the first category, both the damage severity and its proportional area decreases. The signs of corrosion are observed from the distal end to approximately the middle of the taper length. This damage profile was also predicted and observed in the computational results for which the junction keeps its interlock through the experienced cycles. Overall, it appears that the developed FE model can predict the tribocorrosive wear profile at the interface; nevertheless, some discrepancies are noticed in the damage distribution. These discrepancies, together with their possible reasons, will be further discussed in the following discussion.

## 4. Discussion

In this study, an adaptive FE model was developed to simulate the fretting corrosion of the head-neck junction of a hip implant. The results of this study showed that the electrochemical corrosion plays an important role in the amount of the material loss, particularly for junctions that are assembled at lower forces. According to the FE results, for the cases assembled by 2 and 8 kN, chemical wear, after four million cycles, accounted for 50% and 25% of the total material loss, respectively. Hence, ignoring the electrochemical corrosion while investigating the tribocorrosive behaviour of the head-neck junction, as has been done in previous studies, will result in an underestimation of the material loss. This study was developed for a particular taper design comprising of a CoCr/CoCr material combination with a distal angular mismatch of 0.01°. For such a design, the FE results showed that by increasing the assembly force from 2 to 4 kN, the total material loss decreases by 91% after four million cycles of gait loading. However, increasing the assembly force from 4 to 8 kN only decreased the total material loss by a further 2%. This suggests that a minimum value of assembly force is required in order to reduce the material loss in the head-neck junction. Interestingly, when the assembly force is increased above this, it has a minimal influence on the material loss. Such a behaviour was previously seen by English et al. [16], although they only considered the mechanical material loss. In their study, the increase of the assembly force from 2 to 4 kN reduced the material loss by 37%, while the increase from 6 to 8 kN reduced the material loss by just 5%, after six million cycles. This is also in agreement with the experimental study, conducted by Bitter et al. [13]. In their study, the wear damage reduced by increasing the assembly force from 2 to 4 kN, while increasing it from 4 to 15 kN slightly worsened the wear damage. Figure 6 shows that during the first one million cycles, the 2 kN case has similar mechanical and chemical material losses to those of the other cases. Above that cycle, the interlock effect significantly reduces in the 2 kN case; therefore, the relative micromotion in the junction significantly increases. This has resulted in an extreme increase in the material loss in the 2 kN case (both mechanically and chemically). The ratio of mechanical wear to chemical wear is a square root function of normal force (Equations (1) and (2)). Hence, for the 2 kN case, with both the loss of the interlock and an extreme reduction of the contact pressure after one million cycles, the mechanical and chemical material losses have very close values. For the other cases, despite the reduction of contact pressure, the taper junctions maintain their interlocks. This has resulted in a gradual increase in the mechanical and chemical material losses, during four million cycles. Hence, for the particular head-neck junction studied, the minimum required assembly force was 4 kN. Increasing the force above this minimum required assembly loading and did not considerably improve the wear behaviour of the junction.

The FE-predicted tribocorrosion patterns were generally compared with a group of retrieved head-neck junctions. Although proving successful in predicting the damage pattern at the interface, there were some discrepancies in both the damage pattern and its severity. These could be related to a number of reasons including the junction geometry, the mismatch angle, the original assembly conditions and the loading history (both the type and magnitude of the load) applied to the junction by the patient.

This study investigated a single taper geometry and material combination with a distal mismatch only; therefore, caution is required when extrapolating these results to other taper designs. By changing the design parameters, the material combination and the number of cycles, the minimum required assembly force is likely to change. Such an assembly force should be able to maintain the taper interlock effect, despite the chemical and mechanical losses. The hardness and friction coefficients were adopted from Maldonado’s work [22]. These coefficients are functions of test parameters such as frequency, sliding distance and contact pressure. More experimental tests are needed to define the hardness and friction coefficients. Although the FE method was verified against some ball-on-disk experiments and compared with some retrieved head-neck junctions, further work is still required to verify the outcomes of this model for complex head-neck geometries. This is beyond the scope of the present work and is in the prospective of the authors to perform some well-controlled tribocorrosion experiments on head-neck junctions.

## 5. Conclusions

In this study, a tribocorrosion algorithm that was previously developed by authors, was used to assess the influence of assembly force on the tribocorrosion of the head-neck junction. The junction possessed a CoCr/CoCr material combination with a distal angular mismatch of 0.01°. Based on the FE results, the following conclusions may be made:

- Electrochemical corrosion plays an important role in the behaviour of the tribocorrosive behaviour of the head-neck junction; ignoring this leads to a major simplification. The results of this study showed that for different cases, chemical wear was in the range of 25–50% of the total material loss, after four million cycles.
- For the particular design used in this study, the minimum required assembly force was 4 kN. The increase of the assembly force from 2 to 4 kN decreased the total material loss by 91%, within four million loading cycles. However, the increase of this force from 4 to 8 kN improved the tribocorrosive behaviour of the junction by just 2%.
- The profile of the micromotion for the 4, 6 and 8 kN cases (which maintained their interlock effect) changed during the process of simulation. For these cases, the maximum micromotion occurred in the superolateral region, and shows an increasing trend.

## Data Availability

The data presented in this study are available on request.
